# Pineal Process and Psychiatric Manifestations : a case report

**DOI:** 10.1192/j.eurpsy.2026.12128

**Published:** 2026-07-31

**Authors:** K. Taleb, B. Hana, A. Loubna, O. Abderazzak

**Affiliations:** Razi Hospital, Salé, Morocco

## Abstract

**Introduction:**

Tumors in the pineal region can originate from structures surrounding the pineal gland (the posterior part of the third ventricle, the aqueduct of Sylvius, the ambient cistern, the tectal plate, the thalamus, the midbrain, the corpus callosum, the dura mater of the tentorial apex), as well as from the pineal gland itself. These tumors are complex, often histologically composite, and present with different clinical and histological characteristics depending on the age of onset.Psychiatric manifestations are common in patients with pineal processes. This association between pineal processes and psychiatric manifestations raises specific pathophysiological, etiological, and therapeutic questions. We propose to address this issue through a clinical case.

**Objectives:**

Highlight the different psychiatric manifestations associated with the pineal process, as well as the appropriate therapeutic management

**Methods:**

We report the case of a 28-year-old patient with a history of diabetes insipidus since childhood, who has been under neurosurgical care since 2021 for the management of a pineal process following the progressive onset of hydrocephalus with behavioral disturbances and insomnia. Given these symptoms, the patient underwent a brain MRI, which revealed a tumor in the pineal region without signs of peripheral extension. Tumor markers were negative in both blood and CSF.

**Results:**

The patient underwent tumor resection with biopsy and intraoperative examination in 2021, revealing a histological type of germinoma. He then received additional chemotherapy over 12 sessions, with regular follow-up in neurosurgery through physical examination and MRI every six months for the first two years, then once a year. The most recent follow-up MRI showed regression of the process without hydrocephalus. The patient was referred to psychiatry for the management of apathy, indifference with behavioral disturbances, a tendency to wander, delusional verbalizations,

attention and memory disturbances, and insomnia. The psychiatric interview revealed a persecutory delusional syndrome centered on his surroundings, a hallucinatory syndrome with auditory hallucinations, and impaired judgment and insight. The patient was prescribed Aripiprazole 10 mg/day and Trazodone 25 mg/day with good progress.

**Conclusions:**

There are well-defined links between the pineal process and psychiatric manifestations. A better understanding of these links could shed light on the etiopathogenesis of psychiatric symptoms, ensuring better patient care and improving their quality of life.

**Disclosure of Interest:**

None Declared

